# Fermented Dairy Products as Precision Modulators of Gut Microbiota and Host Health: Mechanistic Insights, Clinical Evidence, and Future Directions

**DOI:** 10.3390/foods14111946

**Published:** 2025-05-29

**Authors:** Yuan Gao, Yanyan Liu, Tingting Ma, Qimeng Liang, Junqi Sun, Xiaomeng Wu, Yinglong Song, Hui Nie, Jun Huang, Guangqing Mu

**Affiliations:** 1 School of Biological and Chemical Engineering, Zhejiang University of Science and Technology, Hangzhou 310023, China; 2 Dalian Probiotics Function Research Key Laboratory, School of Food Science and Technology, Dalian Polytechnic University, Dalian 116034, China; 3 Department of Agricultural, Food and Nutritional Science, University of Alberta, Edmonton, AB T6G 2P5, Canada; 4 Guangxi Key Laboratory of Health Care Food Science and Technology, Hezhou University, Hezhou 542899, China

**Keywords:** fermented dairy products, gut microbiota modulation, postbiotics, precision nutrition, host–microbiome interactions

## Abstract

Dairy products—encompassing yogurt, kefir, cheese, and cultured milk beverages—are emerging as versatile, food-based modulators of gut microbiota and host physiology. This review synthesizes mechanistic insights demonstrating how live starter cultures and their fermentation-derived metabolites (short-chain fatty acids, bioactive peptides, and exopolysaccharides) act synergistically to enhance microbial diversity, reinforce epithelial barrier integrity via upregulation of tight-junction proteins, and modulate immune signaling. Clinical evidence supports significant improvements in metabolic parameters (fasting glucose, lipid profiles, blood pressure) and reductions in systemic inflammation across metabolic syndrome, hypertension, and IBS cohorts. We highlight critical modulatory factors—including strain specificity, host enterotypes and FUT2 genotype, fermentation parameters, and matrix composition—that govern probiotic engraftment, postbiotic yield, and therapeutic efficacy. Despite promising short-term outcomes, current studies are limited by heterogeneous designs and brief intervention periods, underscoring the need for long-term, adaptive trials and integrative multi-omics to establish durability and causality. Looking forward, precision nutrition frameworks that harness baseline microbiota profiling, host genetics, and data-driven fermentation design will enable bespoke fermented dairy formulations, transforming these traditional foods into next-generation functional matrices for targeted prevention and management of metabolic, inflammatory, and neuroimmune disorders.

## 1. Introduction

Over the past few decades, urbanization and globalization have changed our diets. People now eat more ultra-processed foods that are high in saturated fats, simple sugars, emulsifiers, and chemical additives. At the same time, antibiotics and proton pump inhibitors are used more often, which disrupts the gut microbiota, causing dysbiosis [1]. Dysbiosis contributes to chronic diseases such as inflammatory bowel disease, obesity, type 2 diabetes, and cardiovascular disease [2]. Fermented dairy products—yogurt, kefir, cheese, and cultured milk beverages—offer a culturally accepted, food-based approach to restore microbial balance. Beyond delivering live probiotic strains (e.g., *Lactobacillus*, *Bifidobacterium*, *Streptococcus*), these matrices are rich in postbiotic compounds—including short-chain fatty acids (SCFAs), bioactive peptides, exopolysaccharides, and bacteriocins—generated during fermentation [3,4]. The dairy matrix helps probiotic microbes survive by buffering gastric acid, providing prebiotic substrates that promote their engraftment, and supplying nutrients such as caseins, whey proteins, calcium, and phospholipids. These nutrients and microbial metabolites work together to strengthen the gut’s mucosal defenses. SCFAs produced in situ engage G-protein-coupled receptors, such as free fatty acid receptor 2/3(FFAR2/3), upregulate tight-junction proteins (ZO-1, occludin), and modulate immune signaling via TLR2/4, thereby strengthening barrier integrity and attenuating inflammation [5,6].

**Figure 1:**
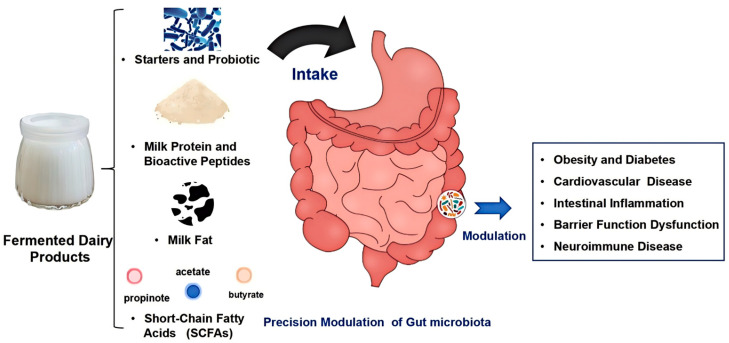
Overview of fermented dairy products as precision modulators of gut microbiota and host health.

Emerging clinical and preclinical evidence demonstrates that regular intake of fermented dairy enhances gut microbial diversity, elevates fecal and systemic SCFA levels, downregulates pro-inflammatory cytokines, and improves metabolic biomarkers in dysbiosis-associated conditions (Figure 1). Meta-analyses report significant reductions in fasting glucose, LDL cholesterol, and systemic CRP following yogurt or kefir interventions, with effect sizes influenced by strain composition, dose, and host genotype [7,8]. To fully realize the therapeutic potential of fermented dairy products, future research will integrate high-resolution omics (metagenomics, metabolomics, and transcriptomics) with well-designed randomized controlled trials across diverse dietary backgrounds. Precision nutrition frameworks, incorporating baseline microbiota enterotypes, host genetic markers (e.g., FUT2 secretor status), and machine learning predictive models, will enable tailored fermented dairy formulations that optimize engraftment, metabolite production, and long-term health outcomes [9,10].

The numbers in the figure indicate the references of this article.

Recent years have witnessed significant advances in characterizing the capacity of fermented dairy to enrich beneficial microbial taxa, elevate short-chain fatty acid production, and reinforce epithelial barrier function. However, existing studies are often limited by short intervention durations, heterogeneous fermentation parameters, and insufficient integration of multi-omics and personalized host factors, which together impede mechanistic clarity and translational application. This review provides a comprehensive summary of how fermented dairy products regulate the gut microbiota and explains the underlying molecular mechanisms. It also evaluates clinical evidence in metabolic, cardiovascular, and immune health and outlines key challenges and future directions for precision-driven nutritional interventions.

## 2. The Role of Starters and Metabolites

### 2.1. Direct Actions of Starters

#### 2.1.1. Stress Tolerance and Colonization

Fermented dairy-derived microbes must survive the stomach’s low pH and the small intestine’s bile salts to reach the colon alive [11,12]. To counteract acid stress, *Lactobacillus*, *Bifidobacterium*, and *Streptococcus* strains utilize F_1_F_0_-ATPase proton pumps, produce ammonia via amino acid deamination, and remodel membrane lipids—enriching cyclopropane and saturated fatty acids—to stabilize intracellular pH and reduce proton influx [11,13]. Upon duodenal entry, bile salt hydrolase (BSH) enzymes deconjugate and detoxify bile acids, while exopolysaccharide capsules and cell-surface proteins shield membranes from bile-induced disruption [12,14]. These intrinsic mechanisms, coupled with adaptive stress responses such as chaperone upregulation, permit a fraction of ingested cells to adhere transiently to the mucosal epithelium and resist peristaltic washout [15].

Once they traverse gastrointestinal barriers, fermented dairy microbes modulate gut ecology via direct pathogen exclusion and indirect metabolic interactions [12]. They competitively consume host-derived glycans and dietary fibers, occupy epithelial adhesion sites, and secrete antimicrobial metabolites—lactic acid, bacteriocins, and hydrogen peroxide—lowering luminal pH to inhibit pathogens such as *Escherichia coli* and *Clostridioides difficile* [12,16]. Concurrently, fermentation of residual oligosaccharides generates short-chain fatty acids (acetate, propionate, butyrate; SCFAs) which fuel colonocytes, enhance tight-junction assembly, and promote mucus production, reinforcing barrier integrity [16,17]. Cross-feeding interactions—where lactic acid produced by lactic acid bacteria (e.g., *Lactobacillus*, *Streptococcus*, *Bifidobacterium*) is converted by specialized anaerobes (e.g., *Anaerostipes* spp., *Eubacterium hallii*, *Coprococcus catus*) into anti-inflammatory butyrate—further diversify metabolic outputs and support a resilient eubiotic community [11]. In these lactate-utilizing bacteria, lactic acid serves as an electron sink for NAD⁺ regeneration during fermentative metabolism. Moreover, residual lactose from fermented dairy products can reach the colon and contribute additional substrates for SCFA production [11]. Colonization dynamics vary by strain: traditional starters such as *Streptococcus thermophilus* and *Lactococcus lactis* survive transit only briefly, with their populations declining rapidly once feeding stops [18]. Probiotic strains such as *Lactobacillus rhamnosus* GG (LGG), *Bifidobacterium animalis* subsp. *lactis* BB12, and *Lactobacillus plantarum* WCFS1 can adhere in the gut for days to weeks or even establish residency for months due to their superior adhesion proteins and carbohydrate utilization loci [18,19]. Combining transient starters with persistent probiotics in complex dairy matrices thus delivers both immediate metabolic stimulation and sustained microbial modulation, providing a blueprint for designing tailored interventions that consider host genetics, diet, and health status [11,15].

#### 2.1.2. Competitive Inhibition

Overlapping ecological strategies underlie the competitive inhibition exerted by probiotic strains in fermented dairy against enteric pathogens. First, ecological niche pre-emption deprives pathogens of essential resources and adhesion sites. Probiotics such as *Lactobacillus* and *Bifidobacterium* species outcompete *Escherichia coli* and *Salmonella* for host-derived glycans and dietary fibers, thereby establishing “colonization resistance” [20]. Through high-affinity binding to mucin receptors (e.g., MUC2) and occupation of epithelial niches, they block pathogen adhesion and deplete local iron and carbohydrate pools, suppressing invader growth [21].

Fermentation-derived targeted antimicrobial secretion further amplifies pathogen suppression. Lactic acid bacteria produce diverse bacteriocins—nisin, plantaricins, reuterin, and pediocins—with strain-specific spectra. Nisin from *Lactococcus lactis* forms membrane pores and, in ripened cheddar with bacteriocin-producing cultures, reduces *E. coli* O157:H7 counts by ≥2 log units over 60 days [22,23]. Reuterin from *L. reuteri* exhibits broad antimicrobial activity, including inhibition of quorum-sensing-dependent biofilm formation by *Salmonella* spp. [24]. Glycoproteins and postbiotic peptides (e.g., from *L. casei* Shirota) disrupt *Clostridioides difficile* biofilms by >60% in colonic mucus models [20].

These dual mechanisms act synergistically to reshape gut ecosystems. In murine colitis models, administration of *L. reuteri* KUB-AC5 yielded both ecological exclusion and direct antimicrobial effects, significantly attenuating *Salmonella*-induced inflammation and pathogen burdens [25]. Clinically, daily kefir intake (300 mL/day) reduced fecal *E. coli* by 40% and decreased serum zonulin by 18% in IBS patients (*p* < 0.01), reflecting enhanced barrier integrity [26]. Future studies should quantify strain-specific bacteriocin production kinetics, investigate multi-strain synergies under varied fermentation conditions, and assess long-term probiotic interventions on gut health and disease prevention.

### 2.2. Indirect Modulation via Microbial Metabolite

#### 2.2.1. Short-Chain Fatty Acids (SCFAs)

Fermentative activity of dairy-associated *Lactobacillus*, *Bifidobacterium,* and *Streptococcus* strains yields millimolar concentrations of acetate, propionate, and butyrate in fermented matrices [27,28]. Water kefir—a non-dairy, mildly alcoholic and acidic fermented drink made by incubating sugar solutions (e.g., sucrose, glucose, fructose) and dried fruits (e.g., figs, dates) with dextran-rich grains hosting a symbiotic culture of lactic acid bacteria, acetic acid bacteria, and yeasts—produced ≈ 48.3 mM acetate and ≈ 13.8 mM propionate after 6 h and ≈ 6.8 mM butyrate after 24 h during in vitro fermentation [28,29]. SCFAs are key mediators of host–microbe crosstalk, modulating immune, metabolic, and epithelial functions via receptors such as FFAR2/3 [29,30]. The resulting drop in luminal pH to ~5.5–6.0 selectively inhibits acid-sensitive pathogens (e.g., *Escherichia coli*, *Clostridioides difficile*) while promoting acid-tolerant commensals such as *Faecalibacterium prausnitzii* and *Roseburia* spp. [31,32].

**Figure 2:**
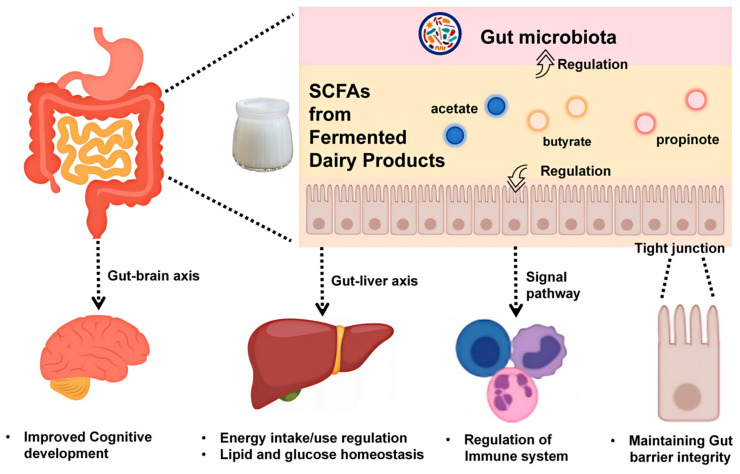
Overview of short-chain fatty acids (SCFAs) from fermented dairy products as precision modulators of gut microbiota and host health.

Animal and human studies demonstrate dose-dependent SCFA elevations following fermented dairy intake, with increased acetate, propionate, and butyrate correlating with enhanced barrier integrity and reduced endotoxemia [27,33]. In rodent colitis models, kefir supplementation elevated proximal colonic SCFAs, upregulated tight-junction proteins (e.g., ZO-1), and decreased systemic lipopolysaccharide (LPS) levels [33]. Propionate, acting via FFAR3 on immune cells, suppresses interleukin 6 (IL-6) and tumor necrosis factor-α (TNF-α) secretion [34,35]. Moreover, combining butyrate-enriched fermented milk reduced colonic nuclear factor kappa-light-chain-enhancer of activated B cells (NF-κB) activation in murine models, highlighting synergistic anti-inflammatory effects [31,36]. These findings underscore the importance of strain specificity, fermentation parameters, and dosage in tailoring SCFA-mediated health outcomes [29,30]. Taken together, these studies demonstrate that SCFAs generated during dairy fermentation exert precise, dose-dependent effects on gut microbial communities and host physiology (Figure 2).

#### 2.2.2. Bioactive Peptides

Bioactive peptides in fermented dairy originate primarily from microbial proteolysis and enzymatic hydrolysis of milk proteins, notably caseins and whey. During fermentation, lactic acid bacteria release proteases that free tripeptides like Ile–Pro–Pro (IPP) and Val–Pro–Pro (VPP) from β- and κ-casein [37,38]. These peptides inhibit angiotensin-converting enzyme (ACE) at low micromolar concentrations, similar to drug-based ACE inhibitors [37,38]. Randomized controlled trials of fermented milk enriched with IPP and VPP have shown small but significant drops in systolic blood pressure in people with hypertension or prehypertension, though the effects depend on the peptide dose and study design [37,39]. Beyond the canonical lactotripeptides, αs1-casein f(24–32) and β-casein f(193–209) released in high-protein yogurts also inhibit ACE and exhibit opioid-like activities that may influence gut motility [40].

In addition to antihypertensive effects, fermented dairy-derived peptides encompass immunomodulatory and antioxidant functionalities. Lactoferricin, a 25-residue peptide produced by proteolysis of lactoferrin, scavenges reactive oxygen species and inhibits pathogenic bacteria, contributing to mucosal protection [41]. κ-Casein fragments generated by *Streptococcus thermophilus* enhance IL-10 secretion in dendritic cells, thereby promoting regulatory T-cell differentiation and attenuating inflammatory responses [42]. Soluble proteins p40 and p75 secreted by LGG preserve Caco-2 tight-junction integrity under cytokine-induced stress via ERK1/2 and PKCβ1 signaling pathways, hindering paracellular permeability increases [43,44]. Peptide yield and spectrum depend critically on fermentation conditions—strain selection, substrate composition, pH, and incubation duration. Co-fermentation with prebiotic fibers such as inulin can double the release of specific peptides like lactoferricin, while controlled acidification (pH 4.0–4.5) maximizes tripeptide stability [37,45]. Fermented dairy also produces “postbiotic” peptides and small molecules like γ-aminobutyric acid, which may reduce anxiety and influence the gut–brain axis [41]. Future studies should combine proteomic and peptidomic analyses with clinical trials, optimize starter cultures and substrates, and clarify how microbes and hosts interact to absorb these peptides and trigger their effects.

### 2.3. Remodeling of Microbial Community Structure

Long-term consumption of fermented dairy products induces profound structural remodeling of the gut microbiota, characterized by enhanced microbial diversity, optimized ecological balance, and functional specialization. In a randomized controlled trial of eight healthy adults (two women, six men; ages 18–54), eating at least 250 g of yogurt daily for 42 days increased their gut microbial diversity—shown by higher Shannon index values and greater *Lactobacillus* abundance—demonstrating that regular yogurt consumption can reshape community structure [46]. In professional female soccer players, 28 days of kefir (200 mL/day) raised the microbial diversity (Shannon and Chao1 indices) and abundance of *Akkermansia muciniphila* and *Faecalibacterium prausnitzii*, which correlated with improved athletic performance variables [47]. In adults with metabolic syndrome, 12 weeks of daily kefir (180 mL/day) raised the relative abundance of *Actinobacteria* (*p* = 0.023), and improved favorable effects on some metabolic syndrome parameters [48]. A previous meta-analysis indicates that drinking yogurt exerts its effect by regulating the metabolism of the gut microbiota and can indirectly reduce the incidence of type 2 diabetes [49]. This remodeling underscores the dynamic interplay between exogenous strains and resident communities.

Yogurt is arguably the most common and ideal probiotic carrier, with strain-specific interventions further refining community composition. Supplementation with *Bifidobacterium animalis* subsp. *lactis* BB-12 (9 × 10^7^ CFU/kg·bw) for 8 weeks resulted in selective inhibition of the growth of *Prevotella* and a decrease in the growth of *Clostridium*, *Blautia*, and *Bacteroide,* ameliorating obesity in high-fat-diet-fed rodent models [50]. Conversely, *Lactobacillus casei* DG^®®^ (2.4 × 10^7^ CFU/day, 8 weeks) preferentially enhanced alpha and beta diversity in ileal pouch microbiota and induced protective changes in pouchitis patients [51]. Yogurt with inactivated probiotics can also benefit gut health. In a double-blind, placebo-controlled trial, drinking heat-treated milk fermented with *Lactobacillus helveticus* CP790 lowered gut *Desulfobacterota* levels, eased constipation (better stool consistency and less straining), and improved mood states [52].

Interindividual variability modulates these effects. A study evaluated how yogurt consumption affects interindividual variation in gut microbiota community structure (β-diversity) by analyzing 16S rRNA and whole-genome metagenomic data from 1004 TwinsUK cohort participants [53]. The results indicated that yogurt had a negligible effect on the overall community structure compared to other host and environmental factors [53]. Recent studies have indicated that intestinal physiological parameters (such as gastrointestinal transit time and pH value) are also significant factors determining the differences in individual microbiota composition. These parameters have a substantial impact on the composition of the microbiota and the production of metabolites [54].

Moreover, ethnicity and habitual diet influence remodeling trajectories. A study conducted in the United States has revealed that the abundances of at least 12 genera/tribe-level microorganisms vary reproducibly among different ethnic groups [55]. These microbial communities often form synergistic clusters and are highly correlated with genetic variations [55]. Another study has found that a higher Healthy Eating Index is associated with an increased ratio of butyric acid/(acetic acid + propionic acid) in feces, suggesting that the overall quality of diet can regulate the relative abundance of different SCFAs [56]. Compared with single food or nutrients, overall dietary patterns (such as the Mediterranean diet and plant-based diet) have a more extensive and sustainable promoting effect on the diversity of the gut microbiota and the production of SCFAs [57].

Future research should leverage shotgun metagenomics and metabolomics to map strain–dose synergies, elucidate host–microbe metabolic networks, and tailor fermented dairy formulations to individual microbial and genetic profiles. Such precision nutrition approaches promise to optimize community-targeted interventions, harnessing the full potential of fermented dairy to restore eubiosis and improve metabolic health.

## 3. Clinical Evidence of Health Benefits

Fermented dairy products, such as kefir, yogurt, and cheese, offer a range of health benefits supported by emerging clinical evidence. These products can play a significant role in managing obesity, diabetes, and cardiovascular disease, mitigating intestinal inflammation and barrier function dysfunction, as well as modulating the neuroimmune system through the gut–brain axis. Table 1 further details the role of fermented dairy products in the gut microbiota and host health in clinical models, providing more specific clinical findings on how these products impact gut microbiota and host health.

### 3.1. Obesity, Diabetes, and Cardiovascular Disease

Emerging clinical data support kefir as an effective adjunct in glycemic management. A clinical study involving 42 adult men newly diagnosed with type 2 diabetes mellitus (T2DM) assessed the effects of kefir consumption on glycemic control [70]. Participants were randomly assigned to two groups: one group received metformin alone, while the other group received metformin in conjunction with a daily intake of one cup of kefir over a 10-week period [70]. The study found that the group consuming kefir alongside metformin experienced a significant reduction in both fasting blood glucose levels and HbA1c values compared to the group receiving only metformin (*p* < 0.05) [70]. These shifts correlate with enhanced insulin sensitivity and upregulated Glucose Transporter Type 4 (GLUT4), an insulin-regulated glucose transporter, translocation in peripheral tissues, underscoring kefir’s role in microbiota-mediated glycemic control [58]. In a sex- and age-stratified cross-sectional study of Chilean adults using a validated online survey, higher dairy product consumption, especially cheese consumption, was significantly linked to a lower obesity risk (adjusted OR 0.70; 95% CI 0.51–0.96; *p* < 0.05) [71]. A meta-analysis of six randomized controlled trials (n = 323) demonstrated that consuming kefir for 4–12 weeks significantly reduced fasting blood glucose (WMD = −10.28; 95% CI: −16.53 to −4.02; *p* = 0.001) and lowered serum insulin (WMD = −2.87; 95% CI: −3.96 to −1.78; *p* < 0.00001), although the decrease in hemoglobin A1c (HbA1c)—an important indicator for measuring blood sugar control—did not reach statistical significance (*p* = 0.08) [72].

Similar findings were observed in metabolic syndrome cohorts. Emerging evidence suggests that kefir consumption may have beneficial effects on lipid profiles in patients with metabolic syndrome. In a randomized controlled trial involving 62 individuals, participants consumed either 180 mL of kefir or unfermented milk daily for 12 weeks [59]. Among those with baseline LDL-C levels above 130 mg/dL, the kefir group experienced reductions of 7.6% in LDL-C and 5.4% in apolipoprotein B, although these changes were not statistically significant compared to the milk group [59]. Additionally, apolipoprotein A1 levels increased by 3.4% in the kefir group, while they decreased by 2.4% in the milk group (*p* = 0.03) [59]. In a randomized controlled trial involving 48 patients with metabolic syndrome, participants were evenly assigned to consume either kefir or curd daily for 12 weeks [60]. The group receiving kefir showed a significant reduction in fasting blood glucose levels, from 95 ± 9 mg/dL to 83 ± 8 mg/dL (*p* < 0.05), while no significant difference in HbA1c levels was observed between the two groups [60]. Probiotic-enriched yogurt also exerts dose-dependent metabolic benefits. In controlled trials, 300 g/day of yogurt containing *Lactobacillus acidophilus* La5 and *Bifidobacterium lactis* Bb12 for six weeks lowered LDL cholesterol by 7.45% (*p* < 0.05) and total cholesterol by 4.54% versus conventional yogurt, while improving atherogenic indices [73]. Consistent with these lipid effects, probiotic yogurt has been shown to reduce the LDL/HDL ratio from 3.13 ± 1.00 to 2.07 ± 0.71 (*p* = 0.016), although changes in triglycerides and total cholesterol were non-significant [61]. Broadly, a meta-analysis underscores that probiotic dairy can improve glycemic and lipid endpoints, with stronger effects in poorly controlled or insulin-resistant individuals [62]. Another meta-analysis of twelve RCTs (total n = 684) showed that daily probiotic supplementation in type 2 diabetes reduced C-reactive protein by 1.34 mg/L (95% CI −1.76 to −0.92; *p* < 0.00001), reflecting attenuated systemic inflammation [74].

Mechanistically, fermented dairy products deliver multiple bioactive effectors. Fermented dairy products confer cardioprotective benefits through a constellation of bioactive compounds that modulate lipid metabolism, vascular tone, and inflammatory signaling. Conjugated linoleic acid (CLA), naturally enriched in yogurt and kefir, exerts hypocholesterolemia effects by inhibiting HMG-CoA reductase and upregulating hepatic LDL receptors [75,76]. Research has demonstrated that SCFAs, particularly butyrate, exert anti-inflammatory effects by activating G-protein-coupled receptors such as GPR43 on endothelial cells. This activation leads to the inhibition of the NF-κB signaling pathway, resulting in decreased expression of pro-inflammatory cytokines and adhesion molecules [77]. SCFAs such as butyrate—produced by both starter cultures and cross-feeding resident microbes—bind GPR43 on endothelial cells to suppress NF-κB signaling, yielding 10–25% reductions in serum CRP in long-term fermented dairy consumers [78]. Meanwhile, a meta-analysis of 33 randomized controlled trials reported a significant reduction in LDL cholesterol levels (95% CI −0.25 to −0.11; *p* < 0.001) following CLA-enriched dairy consumption [79].

Milk consumption usually has no major effect on CVD risk. However, studies suggest that fermented dairy products (e.g., yogurt, cheese) may lower CVD risk and offer cardiovascular benefits [80]. In the multinational PURE cohort, people who ate more than two servings of dairy per day had a lower risk of major cardiovascular events (HR 0.84, 95% CI 0.75–0.94; *p* = 0.0004), cardiovascular death (HR 0.77, 95% CI 0.58–1.01; *p* = 0.029), and stroke (HR 0.66, 95% CI 0.53–0.82; *p* = 0.0003). Milk and yogurt intake were also linked to these benefits [81]. On the other hand, in a study of 150 healthy adults, daily consumption for 30 days of a fermented dairy product containing *Bifidobacterium animalis* subsp. *lactis*, *Streptococcus thermophilus*, and *Lactobacillus delbrueckii* (10^7^ CFU/g) led to an overall increase in gut microbial diversity and a marked enrichment of *Slackia isoflavoniconvertens* and *Adlercreutzia equolifaciens*—species capable of converting dietary isoflavones into equol, a bioactive compound with potential hormonal and cardiovascular benefits [63]. In Russian adults with obesity and hypertension, cheese enriched with the probiotic *Lactobacillus plantarum* TENSIA (DSM 21380) significantly alleviated metabolic syndrome symptoms, as reflected by reductions in BMI (*p* = 0.031) and arterial blood pressure (*p* = 0.0640) [64]. In a two-sample Mendelian randomization analysis of GWAS data, genetically predicted cheese intake was causally associated with lower risks of multi-vascular atherosclerosis (coronary, peripheral, and other vascular AS) and a range of atherosclerotic cardiovascular disease outcomes—including coronary artery disease, angina pectoris, myocardial infarction, heart failure, total ischemic stroke, peripheral artery disease, and cognitive impairment [82]. A combined analysis of China Kadoorie Biobank and UK Biobank data, plus an updated meta-analysis, found that higher total dairy intake was linked to a 3.7% lower risk of new CVD and a 6% lower risk of stroke [83]. These effects were mainly driven by cheese and low-fat dairy. However, whole milk was associated with a 9% higher Coronary Heart Disease (CHD) risk in China, while in Britain the associations were protective [83]. A meta-analysis of 15 prospective studies found that high versus low cheese consumption was inversely associated with risks of total CVD (RR 0.90; 95% CI 0.82–0.99), CHD (RR 0.86; 95% CI 0.77–0.96), and stroke (RR 0.90; 95% CI 0.84–0.97), with a nonlinear dose–response relationship showing maximal risk reduction at approximately 40 g/day of cheese [84].

Host genetics modulate probiotic response (FUT2 secretors display higher baseline bifidobacterial diversity and greater enrichment after fermented dairy intake), underscoring the need for personalized efficacy [85]. Meanwhile, pairing transient starter cultures (e.g., *Streptococcus thermophilus*) with persistent probiotics (e.g., LGG) in complex matrices like kefir delivers both immediate metabolic modulation and sustained microbiota remodeling. Future work should therefore refine strain–dose combinations, elucidate host genetic modifiers, and integrate precision nutrition approaches—including host genotyping, metagenomics, metabolomics, and vascular imaging—to optimize fermented dairy formulations for metabolic health and maximal cardiovascular risk reduction.

### 3.2. Intestinal Inflammation and Barrier Function Dysfunction

Probiotic-enriched fermented dairy formulations have demonstrated clear benefits in mitigating intestinal inflammation and restoring epithelial integrity, particularly in inflammatory bowel disease (IBD) (Table 1). In Dextran Sulfate Sodium (DSS)-induced colitis models, LGG-fermented milk attenuated histopathological scores and preserved colon length, effects attributed to LGG-derived proteins p40 and p75 that activate epithelial EGFR/Akt signaling and inhibit cytokine and oxidative stress-induced tight-junction disruption [86]. Another protein HM0539 secreted by LGG can downregulate the TLR4/MyD88 axis and subsequent NF-κB activation, thereby inhibiting the production of pro-inflammatory mediators in colonic tissues and demonstrating significant anti-inflammatory potential [87]. Traditional fermented products such as kefir further enrich *Akkermansia muciniphila*, a mucin-degrading commensal that enhances mucosal barrier function and reduces colonic inflammation and intestinal barrier dysfunction [26,88].

Short-chain fatty acids (SCFAs)—predominantly acetate, propionate, and butyrate—are key mediators of intestinal barrier reinforcement following fermented dairy intake (Figure 2). Butyrate and propionate act as ligands for G-protein-coupled receptors such as GPR43 on epithelial cells, triggering intracellular signaling that leads to histone deacetylase (HDAC) inhibition, increased histone acetylation and transcriptional activation of tight-junction genes (e.g., ZO-1, occludin) [89]. This molecular cascade enhances transepithelial electrical resistance and reduces paracellular permeability, correlating with 20–30% reductions in circulating LPS levels in cohorts with metabolic dysfunction [90]. In vitro and in vivo studies further confirm that butyrate directly upregulates ZO-1 and occludin protein expression in Caco-2 monolayers, while SCFA treatment preserves barrier integrity under inflammatory challenge [91]. Animal models provide mechanistic proof of SCFA-driven barrier repair (Figure 2). Fatty acids extracted from milk fermented by multiple lactic acid bacteria (such as *Streptococcus thermophilus*, *Lactobacillus bulgaricus*, and *Lactobacillus plantarum* A3) alleviate DSS-induced colitis in mice by improving the intestinal microbiota (increasing the abundance of beneficial bacteria like *Akkermansia* and *Lactobacillus*), reducing the expression of IL-6 and TNF-α, and inhibiting JNK phosphorylation in the MAPK signaling pathway to mitigate inflammation [92]. Except from SCFAs, exopolysaccharide (EPS), lactic acid (LA), and fermented whey from fermented dairy products alleviate inflammatory conditions in colon tissues by significantly reducing pro-inflammatory cytokines, enhancing the expression of tight-junction proteins (such as ZO-1, occludin, and claudin-1), and improving intestinal barrier function [93,94].

Translational human data corroborate these findings (Table 1). A study involving 28 irritable bowel syndrome (IBS) patients who consumed Bifidobacterium-containing fermented dairy products daily showed increased intestinal production of SCFAs (including butyrate), reduced abundance of the potentially pathogenic *Bilophila wadsworthia*, and improved IBS symptoms after 11 days of consumption [65]. Moreover, randomized crossover trials using multi-strain probiotic yogurts demonstrated that sustained consumption maintained elevated SCFA production and barrier protein expression during washout phases, suggesting durable effects beyond active intervention [95]. Synergistic interactions between probiotics and postbiotic compounds amplify anti-inflammatory outcomes. Subjects with functional constipation or diarrhea who consumed symbiotic fermented milk containing *Lactobacillus plantarum* ST-III and inulin daily for 14 days exhibited significant increases in fecal *Bifidobacterium* and *Lactobacillus populations*, notable reductions in harmful bacteria (including *Clostridium perfringens* and *Escherichia coli*), and elevated levels of SCFAs and secretory IgA, with concomitant improvements in gut microbiota balance and immune function [66].

Future research should leverage longitudinal human trials and integrate multi-omics—combining metagenomics, metabolomics, and functional readouts—with precision probiotic strategies to optimize SCFA production, delineate strain-specific efficacy, and refine fermentation parameters (strain selection, substrate composition, and processing conditions), thereby developing fermented dairy formulations tailored for maximal intestinal barrier support in IBD and related inflammatory disorders.

### 3.3. Neuroimmune Modulation

Emerging evidence reveals that neuroactive postbiotics and metabolites from fermented dairy exert anxiolytic and anti-neuroinflammatory effects via the gut–brain axis (Table 1). In a murine model, kefir supplementation significantly enhances the animal microbiome by promoting the proliferation of fecal butyrate-producing bacterial taxa, specifically *Lachnospiraceae* and *Lachnoclostridium* [96]. Concurrently, it modulates both fecal microbiota composition and SCFA production—particularly butyrate and propionate—in the colon and brain, thereby regulating the microbiota–gut–brain axis and ultimately improving brain health outcomes [96]. In an autism mouse model, kefir intervention reshaped the gut microbiota, improved immune function, and reduced repetitive stereotyped behaviors, demonstrating its anxiolytic and behavioral regulatory effects through the microbiota–immune–brain axis [97]. In another randomized, double-blind, placebo-controlled clinical trial, after 8 weeks of intervention with specific strains of *Bifidobacterium* and *Lactobacillus* in kefir fermentation, the depression scores of elderly subjects showed significant improvement, but the effects on inflammatory and oxidative stress markers were limited [67]. The underlying mechanism suggests that kefir from different sources can differently affect systemic immunity and the 5-HT signaling pathway in the colon and gut microbiome, thereby regulating reward-related behaviors [67]. This indicates that fermented beverages themselves are complex “postbiotics” [67].

The metabolites of fermented dairy products modulate adaptive immunity with neuroimmune implications. GABA-enriched fermented milks elevate brain and circulating GABA levels, improving mood and cognition in rodent models of aging by reducing oxidative stress, modulating PI3K/AKT/mTOR and GABA_B_-cAMP-PKA-CREB signaling pathway, and reshaping gut microbiota [98,99,100]. In humans, higher consumption of yogurt and cheese correlates with lower anxiety scores measured by the Spielberger State-Trait Anxiety Inventory (STAI) 3 in university students, suggesting translational relevance of fermented dairy-derived GABA [101]. Emerging research reveals that coordinated GABA level fluctuations across gut–brain pathways and microbial community restructuring synergistically influence neuropsychological regulation mechanisms [102].

Fermentation-derived peptides further augment neuroimmune homeostasis. In aging mice, oral administration of tryptophan-tyrosine (WY) peptide (10 mg/kg for 14 days) from fermented dairy products can inhibit LPS-induced microglial inflammatory responses in the hippocampus and improve long-term potentiation (LTP) deficits and cognitive decline, suggesting that this postbiotic peptide segment has anti-neuroinflammatory effects [103]. In a chronic stress mouse model, pretreatment with Pep14/21 derived from *Lactobacillus gasseri* 505-fermented milk could downregulate the expression of HPA axis-related genes and proteins, reduce colonic inflammatory markers, and significantly improve anxiety-like behaviors, revealing the bidirectional regulatory effect of fermented milk-derived peptides on the brain–gut axis [104].

A comprehensive understanding of fermented dairy’s neuroimmune benefits relies on three key approaches: (1) integrating metagenomic, metabolomic, and neuroimaging data to link microbial strains and postbiotics like GABA and SCFAs to brain function [96,101]; (2) optimizing fermentation parameters to enhance neuroactive compounds, such as GABA [103,104]; (3) identifying host genetic and epigenetic factors influencing individual responses [98,99,100]. These strategies lay the groundwork for precision nutrition, translating fermented dairy into targeted neuroimmune interventions.

## 4. Factors Modulating Efficacy

### 4.1. Strain Specificity

The therapeutic efficacy of fermented dairy products hinges on the genetic and functional heterogeneity of constituent probiotic strains. Even within a single species, genome-encoded traits dictate critical survival and colonization capacities. For example, LGG exhibits superior acid resistance and mucus adhesion—mediated by its SpaCBA pili—allowing for recovery in feces up to one week post-administration [105,106]. Comparative transcriptomics under gut-mimicking conditions reveal that *Lactobacillus plantarum* WCFS1 upregulates exopolysaccharide (EPS) biosynthesis genes, enhancing biofilm formation, while *Lactobacillus acidophilus* NCFM prioritizes bile salt hydrolase (BSH) production, conferring bile tolerance via two distinct bsh loci (bshA and bshB) with unique substrate specificities [107,108]. These functional specializations influence not only strain survival but also their capacity to secrete antimicrobial metabolites and modulate host immunity.

Inter-strain interactions within multi-species formulations further refine health outcomes. Synergistic partnerships, such as the well-documented mutualism between *Streptococcus thermophilus* and *Lactobacillus delbrueckii* subsp. *bulgaricus*, exemplify cross-feeding dynamics through which *L. delbrueckii* subsp. *bulgaricus* breaks down milk proteins into peptides and free amino acids and *S. thermophilus* provides folate and formate to stimulate partner growth and enhance metabolite biosynthesis [109,110]. Conversely, competitive or antagonistic dynamics can emerge; certain bifidobacterial strains produce organic acids or bacteriocins that inhibit co-administered lactobacilli, underscoring the necessity of compatibility screening [111]. Traditional kefir consortia, which combine lactic acid bacteria and yeasts, exemplify how engineered microbial communities can broaden antimicrobial spectra and enhance resilience by leveraging multiple metabolic pathways.

Host–microbe compatibility adds an additional layer of personalization. Strain engraftment and functional impact depend on individual factors such as baseline microbiota composition. A randomized controlled trial in healthy subjects involving *L. paracasei* CNCM I-1518, CNCM I-3689, and *L. rhamnosus* CNCM I-3690 found that the overall microbiota α/β diversity did not change significantly [68]. However, significant differences were observed in the colonization abundance of each candidate strain at different doses, as well as their contributions to the functional genome [68]. Whole-genome comparative analysis indicates that different strains of *Streptococcus thermophilus* have genotypic differences in genes associated with yeast polysaccharide, lactose metabolism, and stress tolerance, which may result in their different survival and functional performance in the host [112,113,114]. These varying characteristics lead to different interventions of *S. thermophilus* with varying degrees of influence on the microbiota structure, mucin alterations, and host metabolism, demonstrating that even within the same species, the probiotic effects of different strains are highly differentiated [112,113,114]. Advances in metagenomic profiling and machine learning-based predictive modeling now enable the design of precision probiotic interventions tailored to host–microbiome interactions, paving the way for next-generation fermented dairy therapeutics.

### 4.2. Host Individual Differences

Interindividual variation in baseline gut microbiota composition critically determines the efficacy of fermented dairy-based interventions. Participants with higher microbial α-diversity at baseline consistently exhibit more pronounced responses to probiotic supplementation [115,116]. For example, in a controlled trial of partially hydrolyzed guar gum, “responders” with Shannon indices ≥3 experienced significant symptom relief and microbial shifts, whereas low-diversity individuals showed minimal changes [115]. Although this study focused on dietary fiber, analogous patterns have been documented for probiotics from fermented dairy interventions. In humans, probiotic strains colonize the gut mucosa in a highly individualized site- and strain-dependent manner, shaped by each person’s baseline host and microbial profiles rather than by detecting probiotics in stool [116]. This colonization process elicits only short-lived, personalized shifts in mucosal community composition and gut gene expression [116]. Supplementation of probiotics after antibiotics did not universally restore the microbiome but showed significant host dependence, demonstrating the decisive influence of host factors on gut microbiome [117].

Host genetics add a further layer of personalization, with the FUT2 “secretor” genotype emerging as a key determinant of probiotic engraftment and function. Secretor status is defined by functional α1,2-fucosyltransferase 2 (FUT2) [85]. It governs the expression of fucosylated mucosal glycans that serve as substrates and adhesion sites for commensals [85]. Non-secretors (homozygous for inactivating FUT2 alleles such as rs601338 G428A) exhibit significantly reduced abundance and diversity of *Bifidobacterium* spp., impairing their capacity to metabolize prebiotic oligosaccharides in fermented dairy [85]. The FUT2 secretor genotype is closely related to the abundance of the *Bifidobacterium* community, revealing the shaping effect of mucosal glycosylation as a host genetic determinant on the intestinal microbiota [85]. In both infants and adults, non-secretor status correlates with lower levels of mucosa-associated *Bifidobacterium* and altered carbohydrate metabolism pathways, leading to diminished short-chain fatty acid production and barrier support [118]. These genetic effects extend beyond FUT2: host genetic variation drives microbial phenotypic diversity, and genetic state shapes microbiota composition across multiple taxa and phyl [119]. However, definitive clinical data linking these polymorphisms to fermented dairy outcomes remain limited.

Integrating host–microbe interplay into personalized nutrition hinges on three complementary strategies. First, baseline microbiota profiling—enterotype classification and α-diversity metrics—can identify individuals most likely to benefit from specific fermented dairy formulations [115,116]. Second, embedding host genotyping refines these predictions by revealing mucosal glycosylation and immune responsiveness that govern probiotic and starter engraftment and metabolism [65,66,67,68,85,86,87,88,89,90,91,92,93,94,95,96,97,98,99,100,101,102,103,104,105,106,107,108,109,110,111,112,113,114,115,116,117,118]. Third, predictive modeling using multi-omics datasets (metagenomics, metabolomics, host genomics) and machine learning algorithms will enable dynamic adjustment of strain selection, dose, and matrix composition to sustain microbial shifts and health outcomes [68,107,108]. By uniting these elements, precision fermentation strategies can move from population-wide recommendations to truly individualized interventions that optimize gut ecosystem modulation and clinical efficacy.

### 4.3. Fermentation Process and Composition

Fermentation parameters and product composition critically determine how dairy matrices shape gut microbiota and confer health benefits. Traditional spontaneous fermentation relies on indigenous microbial communities present on raw materials or in the local environment to initiate and drive biochemical transformations [119]. The resulting products exhibit extensive microbial and chemical diversity, but also significant batch-to-batch variability and potential safety concerns if undesirable microbes proliferate [119]. In contrast, industrial fermentation employs defined starter cultures and tightly controlled processing conditions (temperature, pH, oxygen, nutrient feed) to maximize productivity, consistency, and safety [119,120]. The strain diversity within starter cultures dictates functional outputs, as evidenced by multi-species consortia interactions [120]. Specifically, kefir-associated communities composed of *Lactobacillus*, *Leuconostoc*, *Lactococcus*, and yeasts generate a broader array of metabolites (e.g., SCFAs, exopolysaccharides, bioactive peptides), which enhance microbial diversity and intestinal barrier function in both murine and human models [120]. Also, milk matrix composition—fat content, protein structure, and prebiotic oligosaccharides—affects microbial growth and metabolite bioavailability. Fat content is positively correlated with the growth rate of probiotics. High-fat dairy products significantly enhance the fermentation activity of starters [121]. Finally, post-fermentation processing (heat treatment, spray drying) can alter microbial viability and metabolite stability. Pasteurized kefir retains exopolysaccharide-driven IL-10 induction despite reduced viability, demonstrating that non-viable postbiotics remain functional [28].

By harnessing advances in microbial ecology and analytical technologies, we can now deliberately engineer every stage of dairy fermentation to optimize health outcomes. Designing tailored starter communities—combining complementary bacterial and yeast strains—allows us to generate precise blends of SCFAs, bioactive peptides, and polysaccharides [121]. Fine-tuning fermentation conditions (temperature, pH, and time) further directs metabolic pathways toward desired products, while adjusting the milk matrix (fat, protein, and oligosaccharide composition) shapes microbial growth and metabolite availability [119,120]. Finally, judicious post-processing (such as gentle pasteurization or microfiltration) preserves key postbiotics without sacrificing safety or shelf stability [119,120]. This integrated, data-driven approach yields fermented dairy foods that not only capture authentic flavors but also deliver targeted gut-modulating, anti-inflammatory, and metabolic benefits, setting a new standard for functional food design.

## 5. Current Research Limitations and Future Directions

### 5.1. Predominance of Short-Term Effects

Current clinical trials of fermented dairy interventions overwhelmingly employ short-term designs (4–12 weeks), which limits insights into sustained microbiota modulation and long-term health effects. A recent systematic review identified 52 randomized controlled trials (RCTs) of probiotic-fortified dairy matrices versus non-probiotic controls, of which over 80% implemented intervention periods of 8–12 weeks and assessed acute endpoints such as fecal SCFA concentrations, inflammatory cytokines (IL-6, TNF-α), or transient increases in *Lactobacillus* abundance [8]. Although existing studies confirm rapid metabolic and immunological responses to fermented dairy products, they leave unresolved whether microbial community restructuring persists after cessation or how chronic conditions (e.g., metabolic syndrome and IBD) progress under prolonged exposure. Furthermore, the potential for abrupt withdrawal to trigger rebound dysbiosis remains unaddressed in current research frameworks [8].

This reliance on short-term RCTs introduces several critical limitations. First, diet interventions often elicit rapid shifts in gut microbiota that revert toward baseline soon after the intervention ends; reviews of fermented food trials underscore the need for longer-term studies before durable health recommendations can be made [122], and fecal microbiota transplantation studies in ulcerative colitis likewise show that short-term benefits frequently wane without continued treatment [123]. Second, short-term studies overlook adaptive microbe–host dynamics [124]. For example, extended exposure to dairy-derived bacteriocins such as nisin could select for resistant pathogen subpopulations, a risk that remains unassessed in 12-week trials [124]. Third, key microbiome biomarkers like the Firmicutes/Bacteroidetes ratio exhibit substantial intraindividual variability over several months, rendering short-term snapshots unreliable predictors of long-term metabolic outcomes [124].

To move beyond these constraints, future research must embrace longitudinal, adaptive trial designs and integrative biomarker strategies. Extended intervention–withdrawal cycles (e.g., 6-month dosing followed by 3-month washout) will elucidate microbiota resilience and host “metabolic memory” [125]. Multi-omics integration, which combines metagenomic sequencing, targeted metabolomics of SCFAs and bile acids, and host epigenomic profiling such as DNA methylation analysis in immune regulatory loci, enables the comprehensive mapping of sustained regulatory networks activated by fermented dairy consumption [125]. This approach systematically links microbial functional dynamics, metabolite-mediated signaling, and host epigenetic reprogramming to elucidate persistent immune–metabolic adaptations [125]. Finally, real-world dietary contextualization through 12-month observational cohorts in diverse nutritional environments (high-fiber vs. high-fat backgrounds) will determine how habitual consumption patterns shape efficacy [125]. Addressing these design and biomarker gaps is essential to ascertain whether fermented dairy products function merely as transient modulators or can serve as foundational dietary therapies for chronic disease prevention and management.

### 5.2. Unclear Mechanisms: Challenges in Establishing Causal Relationships

Although numerous studies link fermented dairy-induced shifts in gut microbiota to health improvements, establishing direct causality remains challenging. Observational studies and short-term interventions frequently identify microbial–host correlations—such as elevated Lactobacillus levels linked to glycemic improvements or reduced fecal calprotectin—yet these associations cannot clarify causality between microbiota shifts and clinical outcomes or host-mediated effects from dietary bioactive components [126]. For instance, while dysbiosis is strongly tied to metabolic syndrome and inflammatory bowel disease (IBD), cross-sectional analyses remain ambiguous about temporal relationships: microbial disturbances may either trigger pathogenesis or arise as adaptations to disease-driven environmental changes [126]. This limitation underscores the need for longitudinal designs to resolve whether dysbiotic states precede pathology or are its downstream consequences [126]. Similarly, diet-induced increases in SCFAs may reflect microbiota shifts or direct fermentation of dietary fibers by exogenous strains—an ambiguity that confounds mechanistic interpretation [4].

Animal models—germ-free (GF) mice and antibiotic-treated rodents—offer controlled settings to probe causal links but suffer from translational limitations. Due to the absence of gut microbiota, germ-free mice have an underdeveloped immune system, abnormal intestinal structure and function, and restricted metabolic regulation and exhibit anxiety-like behaviors [127,128]. These characteristics make them a powerful tool for studying the interaction between gut microbiota and the host, but also limit their application in simulating human physiological states [127,128]. Experimental models using antibiotic-induced dysbiosis frequently rely on high-dose, broad-spectrum regimens that cause non-selective depletion of microbial communities, creating artifacts that diverge from the gradual, diet-mediated ecological shifts observed in human studies [127,128]. These methodological frameworks frequently overlook essential host factors, such as mucosal immune responses, gut motility dynamics, and hormonal oscillations, that dynamically interact with microbial communities through reciprocal regulatory mechanisms [127,128]. Consequently, the inability to disentangle host–microbe interdependencies compromises causal attribution of health outcomes exclusively to microbial mechanisms.

To address causality gaps in fermented dairy research, integrating multi-omics and systems biology has become essential. Recent advances in multi-omics integration demonstrate that combining metagenomic sequencing with untargeted metabolomic analyses enables systematic mapping of microbial biosynthetic gene clusters to functionally critical postbiotic metabolites, including butyrate and immunomodulatory indole derivatives [129,130]. Concurrently, host-derived multi-layer datasets—spanning transcriptomic profiles and epigenomic modifications such as immune-related DNA methylation patterns—uncover persistent regulatory mechanisms through which microbial communities orchestrate long-term immune and metabolic adaptations in the host [129,130]. To build on these molecular insights, rigorously controlled human crossover trials using standardized fermented dairy formulations that contain fixed strain consortia and defined metabolite outputs can clarify dose–response relationships [69]. These trials can also reveal how stable the microbiota and metabolic profiles remain over time [69]. For instance, a double-blind, placebo-controlled crossover study of fermented milk containing *Bifidobacterium bifidum* YIT 10,347 demonstrated significant improvements in gastrointestinal function and gut metabolite profiles, with benefits persisting through washout periods, underscoring the value of phased intervention–withdrawal designs [69].

Finally, synthetic microbial ecology approaches in germ-free or humanized rodent models allow for causal testing of specific metabolites. Supplementing Crohn’s disease patient microbiota with butyrate-producing bacteria (e.g., *Faecalibacterium prausnitzii* and a mix of six butyrate producers) significantly increased butyrate production and improved epithelial barrier integrity in vitro [131]. The study provides direct evidence of the beneficial effects of butyrate-producing bacteria on gut health [131]. Researchers engineered gut microbiota consortia with and without butyrate-producing strains to move beyond correlations and directly demonstrate that butyrate strengthens the intestinal barrier and blocks pro-inflammatory signaling, thus proving a causal mechanism [131].

### 5.3. Personalized Intervention

Precision nutrition harnessing individual gut microbiota profiles offers a route to maximize the benefits of fermented dairy. Integrating multi-omics (metagenomics, metabolomics, and host genomics) enables the identification of predictive microbiota biomarkers that stratify responders versus nonresponses [132]. For example, Bacteroide-dominant enterotypes have been linked to greater SCFA production after kefir consumption [132]. Recent trials have leveraged baseline microbial diversity and functional gene clusters—such as bile salt hydrolase loci—to predict metabolic improvements from fermented milk [133,134] Technological innovations are crucial for implementing precision fermented dairy interventions in real time. Portable, smartphone-connected nanopore sequencers now allow for weekly gut microbiome profiling at home, enabling adaptive dietary adjustments based on immediate feedback. Moreover, advanced analytical methods such as machine learning, mediation analysis, and mendelian randomization provide actionable algorithms for tailoring fermented dairy regimens [135]. Finally, embedding specific strains of probiotics into individualized prebiotic matrices through 3D printing of functional foods can promote the colonization of probiotics and the generation of metabolites [135].

Despite these advances, key challenges remain. (1) Data interpretation and privacy: Consumers may be overwhelmed by complex microbiome reports, and robust frameworks for data ownership and consent are lacking [133,134,135]. (2) Scaling personalized fermented dairy requires modular bioreactors with real-time control of pH, temperature, and substrate feeds, together with algorithmic nutrition platforms that integrate continuous biodata such as glucose monitoring and microbiota sequencing to provide adaptive dietary feedback [133,134,135]. This sensor–network paradigm enables fermented dairy derivatives to advance beyond generic probiotic formulations, emerging as neuro-metabolically programmed therapeutic matrices specifically engineered for inflammatory dysregulation management in the future.

### 5.4. Multi-Omics Integration

The integration of metagenomics, metabolomics, and transcriptomics provides an unparalleled framework to delineate causal host–microbiota interactions in response to fermented dairy interventions [136,137,138]. Metagenomics yields both taxonomic and functional blueprints by sequencing total microbial DNA and identifying species–specific gene clusters such as those encoding short-chain fatty acid (SCFA) biosynthesis pathways [136,137,138]. Metabolomics complements this by quantifying microbial and host metabolites—SCFAs, bile acids, and indole derivatives—that serve as mediators of cross-kingdom signaling [136,137,138]. Transcriptomics captures dynamic gene expression in host tissues and microbial populations, revealing how dietary postbiotics modulate epithelial and immune responses in real time [136,137,138].

Bioinformatic tools such as QIIME 2 (Quantitative Insights Into Microbial Ecology 2), MOTHUR, and MetaPhlAn (Metagenomic Phylogenetic Analysis) support multi-omics integration, enabling synchronous processing of 16S rRNA gene, shotgun metagenomic, metatranscriptomic, and metabolomic datasets within a unified framework [130]. Machine learning frameworks—ranging from random forests to meta-learning models—are increasingly applied to integrate these high-dimensional datasets, enabling the prediction of personalized responses to fermented dairy based on baseline multi-omics profiles [139]. Despite its promise, multi-omics integration faces challenges: batch effects across platforms, incomplete reference databases for novel microbial genes, and the computational complexity of harmonizing disparate data types [139]. In the future, research priorities should focus on longitudinal sampling to improve temporal resolution, employ spatially resolved omics techniques (such as single-cell transcriptomics and laser microdissection) to decipher cell-type-specific responses, and establish standardized data formats to boost reproducibility. By surmounting these challenges, integrated multi-omics approaches will bring about mechanistic clarity in fermented dairy research, informing precision nutrition strategies that customize formulations according to individual host–microbe ecosystems.

## 6. Conclusions

Fermented dairy products have been consistently shown to modulate gut microbial ecosystems through multiple complementary mechanisms. They enrich beneficial taxa such as *Lactobacillus*, *Bifidobacterium*, and *Faecalibacterium prausnitzii*, increasing community diversity and functional resilience [140]. Their complex matrices deliver live probiotic strains alongside fermentation-derived postbiotics—SCFAs, bioactive peptides, exopolysaccharides, and bacteriocins—that synergistically enhance mucosal barrier integrity via upregulation of tight-junction proteins and activation of immune cells [9,141]. Clinical analysis demonstrates that regular consumption of yogurt or kefir significantly reduces fasting glucose, LDL cholesterol, blood pressure, and systemic inflammatory markers in metabolic syndrome, hypertension, and IBS cohorts, with effect sizes modulated by strain composition and dose [62,65,79,81]. Moreover, fermented dairy interventions enhance SCFA production in vivo, elevating colonic butyrate and propionate levels that fuel colonocytes, thereby dampening pro-inflammatory signaling and reinforcing barrier function in preclinical colitis models [134,142].

The efficacy of fermented dairy products is profoundly influenced by multiple modulatory factors. Genomic and functional heterogeneity among strains dictates survival, adhesion, and metabolite profiles [106,143]. For instance, LGG SpaCBA pili confer superior mucus binding compared to transient starters, while *Streptococcus thermophilus* and yeast consortia in kefir broaden postbiotic spectra through mutualistic cross-feeding [106,143]. Host factors such as baseline enterotype, FUT2 secretor genotype, gastrointestinal transit time, and mucosal glycosylation determine probiotic engraftment and response magnitude, highlighting the importance of microbiota and genetic profiling before intervention in clinical trials [115,116,117,118]. Fermentation parameters (pH, temperature, substrate ratios) and post-processing (heat treatment, microfiltration) further shape peptide yields and SCFA composition, offering levers to tailor functional outputs [9,120,121]. By integrating metagenomics, metabolomics, peptidomics, and transcriptomics, researchers can map strain–dose interactions and host–microbe metabolic networks, creating precision nutrition frameworks that adapt fermented dairy formulations to each individual’s profile [132,133,134].

Despite these advances, critical knowledge gaps persist. Most clinical trials remain short-term (4–12 weeks), limiting insights into the durability of microbial shifts and long-term health outcomes; extended intervention–withdrawal designs and year-long observational cohorts are needed to assess resilience and metabolic memory [8,122,123,124]. Causal mechanisms linking specific postbiotic metabolites to clinical endpoints require validation through engineered consortia in humanized models and rigorously controlled crossover trials with standardized matrices [107,108,109,110,134]. Personalized efficacy demands predictive algorithms integrating baseline multi-omics with host genetics, while scalable production of customized fermented dairy calls for modular bioreactors and real-time microbial monitoring [133,134,135]. Longitudinal multi-omics studies, precision intervention trials, and data-driven fermentation design are needed to transform fermented dairy from a traditional food into targeted therapies for metabolic, inflammatory, and neuroimmune disorders [132,133,134,135].

Building on mechanistic insights and clinical evidence, this review highlights how strain specificity, host genetic background, and tailored fermentation parameters converge to determine the health-promoting potential of fermented dairy. By integrating multi-omics data and precision nutrition frameworks, bespoke formulations can be designed to optimize short-chain fatty acid production, epithelial barrier reinforcement, and the release of immunomodulatory peptides. While short-term trials demonstrate promising metabolic and anti-inflammatory effects, long-term, adaptive study designs are needed to confirm the durability of benefits. Ultimately, coupling data-driven fermentation strategies with personalized host profiling will elevate fermented dairy from traditional foods to next-generation functional matrices for targeted prevention and management of metabolic, inflammatory, and neuroimmune disorders.

## Figures and Tables

**Table 1:** The role of fermented dairy products in the gut microbiota and host health in a clinical model *.

Fermented Dairy Products	Model	Dose	Time of Intervention	Effect	References
Kefir and milk	28 asymptomatic overweight adults	300 mL/day	3 weeks	Effect on the intestinal mucosa: a greater improvement in serum zonulin levels (F = 6.812, η2 = 0.275)	[26]
Three commercially available brands of yogurt, with active probiotics *S. thermophilus*, *L. bulgaricus*, *L. acidophilus*, *L. casei,* and *Bifidobacterium bifidus.*	8 healthy subject volunteers (2 females and 6 males) between the ages of 18 and 54	908 g provided to each subject every 3 to 4 days	42 days	Effect on gut microbiota: modulatory effects on *Lactobacilli* populations and manifested a dose-dependent association with modest microbial diversity enhancement in select cohorts	[46]
Kefir	21 female soccer players aged 18–29 years	200 mL/day	28 days	Effect on gut microbiota: enhanced gut microbiota diversity and significantly boosted the abundance of *Akkermansia muciniphila* and *Faecalibacterium prausnitzii*	[47]
Kefir and unfermented milk	Patients with metabolic syndrome	180 mL/day	12 weeks	Effect on gut microbiota and immune: significantly reduced fasting insulin, HOMA-IR, TNF-α, IFN-γ, and both systolic and diastolic blood pressure, and significantly increased relative abundance of *Actinobacteria*,	[48]
*Bifidobacterium animalis* subsp. *lactis* BB-12 (BB-12)	Human microbiota-associated rats	9 × 10^7^ colony-forming unit (CFU)/kg·body weight (bw)	8 weeks	Effect on gut microbiota: prevented the shift from a healthy to an obese state by preserving the *Prevotella*-dominant enterotype	[50]
*Lactobacillus casei* (*L. casei*) DG^®®^	52 patients undergoing restorative proctocolectomy	24 billion	8 weeks	Immune effect: significantly reduced inflammatory cytokine levels in the pouch mucosa compared to baseline	[51]
Heat-treated *Lactobacillus helveticus* CP790-fermented milk	Healthy Japanese individuals aged 20–59 years	100 mL/day	4 weeks	Anti-anxiety effects: participants had firmer stool consistency, less straining, markedly improved overall mood, and lower depression dejection scores	[52]
Kefir with metformin	42 newly diagnosed diabetic male patients aged from 37 to 65 years	250 mL kefir/day	10 weeks	Antidiabetic effects: significantly reduced fasting blood glucose and glycohemoglobin (HbA1c) levels, as well as increased calcium concentrations accompanied by decreased phosphorus levels	[58]
Kefir or unfermented milk	62 patients with metabolic syndrome	180 mL/day	12 weeks	Immune effect: significantly reduced serum LDL-C and apolipoprotein B levels, lowered systolic and diastolic blood pressure, and significantly decreased serum levels of TNF-α, IL-6, IL-10, IFN-γ, and homocysteine	[59]
Kefir beverage and curd	48 patients with metabolic syndrome	1.6 mL/kg for men or 1.9 mL/kg for women	12 weeks	Cardiovascular protective effect: lowered blood pressure, fasting glucose, LDL-C, non-HDL-C, triglycerides, and oxidized LDL while increasing HDL-C	[60]
Probiotic (containing *Lactobacillus acidophilus* La5 and *Bifidobacterium lactis* Bb12) and conventional yogurt	60 people (23 males and 37 females) with type 2 diabetes and low-density lipoprotein cholesterol (LDL-C) greater than 2.6 mmol/L	300 g/day	6 weeks	Cardiovascular protective effect: a 4.54% reduction in total cholesterol and a 7.45% reduction in LDL-C, and a significantly drop in the total cholesterol/HDL-C and LDL-C:HDL-C ratios,	[61]
Yogurt containing *Lactobacillus acidophilus* La-5 and *Bifidobacterium lactis* Bb-12	44 patients with type 2 diabetes aged 30–60 years old who had low-density lipoprotein cholesterol (LDL-c) ≥100 mg/dL	300 g/day	8 weeks	Cardiovascular protective effect: significantly reduced the LDL-C/HDL-C ration and significantly increased HDL-C levels	[62]
Yoghurt fortified with *Bifidobacterium animalis* subsp. *lactis* BB-12	150 healthy adults	125 mL in the morning and 125 mL in the evening daily	30 days	Effect on gut microbiota: paired comparison of gut microbial content revealed an increase in beneficial bacteria, particularly the Bifidobacterium genus, along with *Adlercreutzia equolifaciens* and *Slackia isoflavoniconvertens*	[63]
Cheese enriched with the probiotic *Lactobacillus plantarum* TENSIA (DSM 21380)	25 adults with obesity and hypertension	50 g/day	3 weeks	Weight loss effect: probiotic cheese helps to reduce BMI and arterial BP values	[64]
A fermented milk product containing dairy starters and *Bifidobacterium animalis*	32 patients with irritable bowel syndrome	125 g/serving twice a day	4 weeks	Effect on gut microbiota: enhanced colonic short-chain fatty acid production and reduced the abundance of the pathobiont *Bilophila wadsworthia*	[65]
A fermented milk containing both probiotics (*Lactobacillus plantarum* ST-III) and prebiotics (inulin)	198 participants (ratio of male and female, 1:1) aged 25–45 years old with functional constipation or functional diarrhea	7 mg/kg *Lactobacillus plantarum* ST-III and 1–1.5% inulin	28 days	Effect on gut microbiota: markedly boosted fecal *Bifidobacteria* and *Lactobacillus* while reducing *C. perfringens* and *E. coli*, rebalancing the gut microbial ecosystem and elevating fecal acetic acid, total SCFAs, and SIgA in participants.	[66]
Kefir fortified with *Lactobacillus helveticus* and *Bifidobacterium longum* and kefir used as placebo	67 elderly men aged over 65	240 cc	8 weeks	Anti-anxiety effects: significantly greater improvements in depression scores alongside a marked increase in total antioxidant capacity	[67]
A multi-strain fermented milk product combining *Lactobacillus paracasei* subsp. *paracasei* CNCM I-1518 and CNCM I-3689 and *Lactobacillus rhamnosus* CNCM I-3690	96 healthy adults	Two doses (1 or 3 bottles)/day	4 weeks	Effect on gut microbiota: probiotic strains were detected only during consumption while overall gut α- and β-diversity remained unchanged, and ZIBR analysis revealed only a few genera exhibiting dose-dependent differential responses to the test product	[68]
Fermented milk containing the probiotic *Bifidobacterium* bifidum YIT 10,347 and *S. thermophilus* YIT 2021	27 subjects with gastric symptoms	100 mL/day	2 weeks, followed by crossover for 3 weeks after a washout period	Weight loss effect: ingestion of the active preparation significantly decreased the average gastric symptoms score per subject by 1.0 at 1 week and 1.1 at 2 week	[69]

* The results of the meta-analysis were not presented.

## Data Availability

The original contributions presented in the study are included in the article. Further inquiries can be directed to the corresponding authors.
